# Neonatal hyperoxia enhances age-dependent expression of SARS-CoV-2 receptors in mice

**DOI:** 10.1038/s41598-020-79595-2

**Published:** 2020-12-28

**Authors:** Min Yee, E. David Cohen, Jeannie Haak, Andrew M. Dylag, Michael A. O’Reilly

**Affiliations:** The Department of Pediatrics, School of Medicine and Dentistry, The University of Rochester, 601 Elmwood Avenue, Box 850, Rochester, NY 14642 USA

**Keywords:** Respiratory tract diseases, Respiratory system models

## Abstract

The severity of COVID-19 lung disease is higher in the elderly and people with pre-existing co-morbidities. People who were born preterm may be at greater risk for COVID-19 because their early exposure to oxygen (hyperoxia) at birth increases the severity of respiratory viral infections. Hyperoxia at birth increases the severity of influenza A virus infections in adult mice by reducing the number of alveolar epithelial type 2 (AT2) cells. Since AT2 cells express the SARS-CoV-2 receptors angiotensin converting enzyme (ACE2) and transmembrane protease/serine subfamily member 2 (TMPRSS2), their expression should decline as AT2 cells are depleted by hyperoxia. Instead, ACE2 was detected in airway Club cells and endothelial cells at birth, and then AT2 cells at one year of age. Neonatal hyperoxia stimulated expression of ACE2 in Club cells and in AT2 cells by 2 months of age. It also stimulated expression of TMPRSS2 in the lung. Increased expression of SARS-CoV-2 receptors was blocked by mitoTEMPO, a mitochondrial superoxide scavenger that reduced oxidative stress and DNA damage seen in oxygen-exposed mice. Our finding that hyperoxia enhances the age-dependent expression of SARS-CoV-2 receptors in mice helps explain why COVID-19 lung disease is greater in the elderly and people with pre-existing co-morbidities.

## Introduction

COVID-19 is an infectious disease of the lung caused by the severe acute respiratory syndrome coronavirus (SARS-CoV-2). As of November 2020, the World Health Organization reported this virus has infected over 50 million people worldwide and killed more than 1 million people (https://covid19.who.int). Common symptoms include fever, cough, fatigue, shortness of breath, and loss of olfactory or gustatory function. While the majority of cases are mild, some people progress into severe acute respiratory distress syndrome, multi-organ failure, thrombosis, and septic shock. The severity of disease and mortality is highest among the elderly and people who have pre-existing lung or heart disease. There is growing evidence that asymptomatic children and young adults with COVID-19 may be at risk for heart disease, inflammatory vascular disease, and stroke^1^. People who were born preterm may be at great risk for COVID-19 because they are already at risk for hospitalization following infection with RSV, rhinovirus, human bocavirus, metapneumovirus, and parainfluenza viruses^2^. Preterm birth is a risk factor for pulmonary vascular disease and heart failure^3,4^ that puts these individuals at further risk for COVID-19. Identifying mechanisms that drive susceptibility to pandemic viral infections like SARS-CoV-2 is therefore of great concern to susceptible individuals and their families.

The severity of COVID-19 is likely to be related to age-related changes in SARS-CoV-2 receptors and how the immune system responds to infection^1^. Emerging evidence indicates high-risk individuals with SARS-CoV-2 have high rates of alveolar epithelial type 2 (AT2) cell infection, suggesting disease severity may be related to higher alveolar expression of the SARS-CoV-2 receptor angiotensin converting enzyme (ACE2) and its co-receptor transmembrane protease/serine subfamily member 2 (TMPRSS2)^5,6^. In fact, a recent meta-analysis of 700 people with predicted COVID-19 co-morbidities found that their lungs expressed high levels *Ace2* mRNA^7^. ACE2 is a zinc containing metalloprotease present at the surface of cells in the lung, heart, intestines, kidneys, and brain. It lowers blood pressure by catalyzing the hydrolysis of the vasoconstrictive molecule angiotensin II to angiotensin (1–7). ACE2 co-precipitates with transmembrane protease/serine subfamily member 2 (TMPRSS2), which hydrolyzes the S protein on coronaviruses, thus enabling viral entry into infected cells^6,8^. Higher expression of these proteins in AT2 cells would theoretically lead to higher rates of infection in the distal lung. Infected AT2 cells produce inflammatory mediators that could contribute to a lethal cytokine storm^9,10^. Because AT2 cells also produce surfactant and serve as adult stem cells for the alveolar epithelium, excessive infection and loss of AT2 cells may compromise alveolar homeostasis and regeneration^11^. In fact, high rates of AT2 infection have been seen in people who have succumbed to H5N1, a highly pathogenic avian strain of influenza A virus^12–14^. Since ACE2 expression increases to control blood pressure, COVID19-related disease may be higher in people with pre-existing cardiovascular disease including those people born preterm^15^. But whether aging or pre-existing lung co-morbidities like preterm birth enhance the severity of respiratory viral infections via changing expression of viral receptors is not yet known.

Since preterm infants are exposed too soon to oxygen, we have been using mice to understand how high levels of oxygen at birth increases the severity of influenza A virus infection in adults. We previously reported how adult mice exposed to hyperoxia (100% oxygen) between postnatal days 0–4 have simplified lungs attributed in part to lower numbers of AT2 cells and disorganized elastin fibers. They also develop persistent inflammation and fibrotic lung disease when infected with influenza A viruses HKx31 (H3N2) or PR8 (H1N1)^16,17^. Neonatal hyperoxia does not enhance primary infection^18^ or clearance^19^ of the virus. Instead, it drives fibrotic lung disease by reducing the number of AT2 cells available for regenerating the alveolar epithelium^20^. Because neonatal hyperoxia reduces the number of AT2 cells, we predicted it would also reduce the expression of ACE2 and TMPRSS2 in the distal lung. Instead, we made the surprising discovery that pulmonary expression of ACE2 and TMPRSS2 increases as mice age and this age-dependent expression can be enhanced by early exposure to hyperoxia. Our findings in mice suggest temporal and spatial changes in expression of SARS-CoV-2 receptors may contribute to the increased severity of COVID-19 seen in the elderly and people with pre-existing co-morbidities, including those born preterm.

## Results

### ACE2 is initially expressed by Club cells and then by AT2 cells as mice age

**Figure 1:**
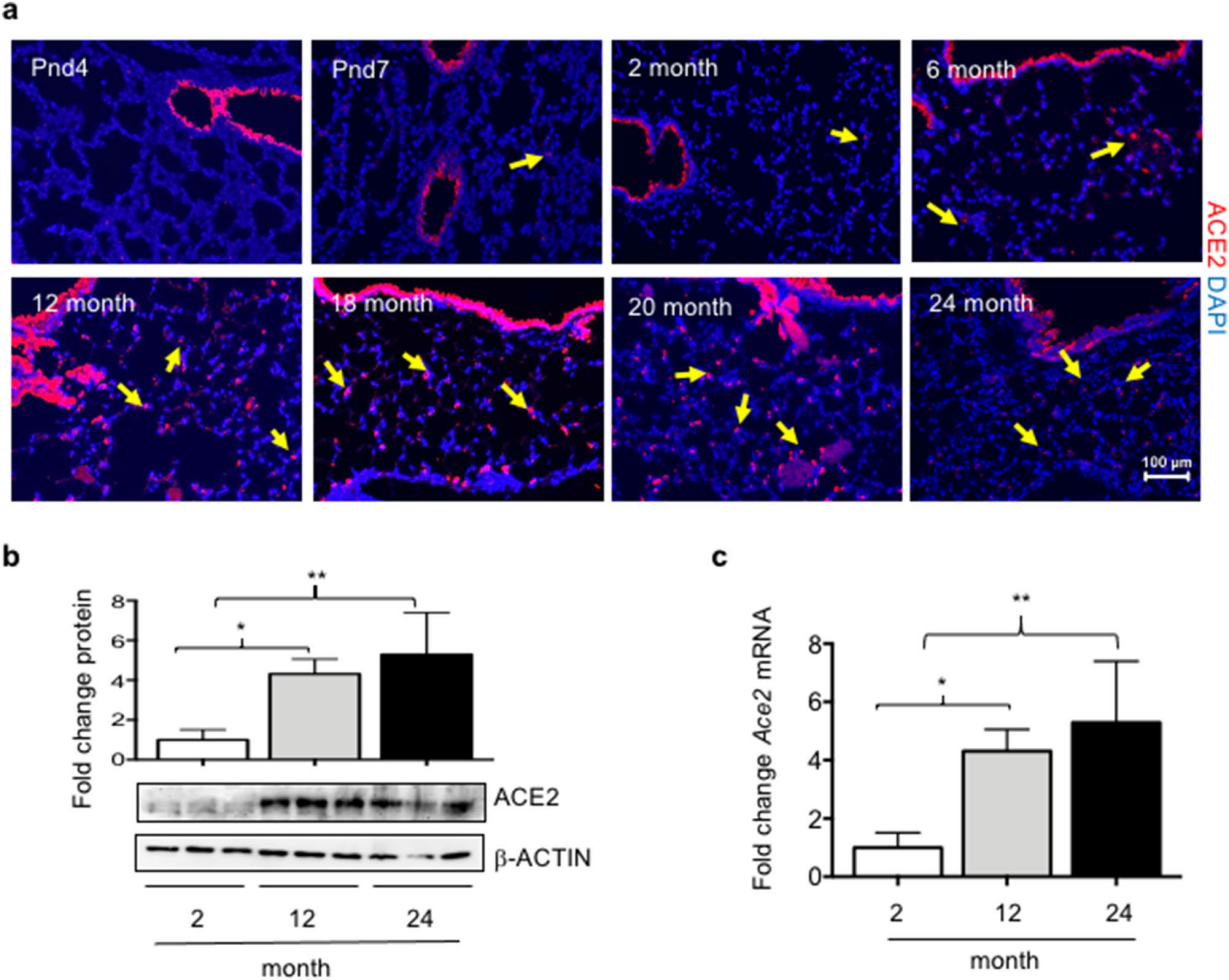
ACE2 expression changes in lung as mice age. (**a**) Lungs harvested from mice of different ages were stained for ACE2 (red) and counterstained with DAPI (blue). ACE2 was detected in airways of all mice and alveolar regions (yellow arrows). Bar = 100 mm. (**b**) Lungs homogenates prepared from 2-month, 12-month, and 24-month-old mice were immunoblotted for ACE2 and β-ACTIN as a loading control. Each lane represents an individual mouse. Band intensity of ACE2 to β-ACTIN was quantified and graphed as fold change relative to 2-month samples. Bars reflect mean ± SD graphed. (**c**) qRT-PCR was used to quantify *Ace2* mRNA in total lung homogenates of 2-month and 24-month-old mice. Data is graphed as the fold change of *Ace2* after normalizing to *18S* RNA from 5 mice per group. Bars reflect mean ± SD graphed as fold change over 2-month values. Statistical significance is comparisons for all pairs using Tukey–Kramer HSD test, with *P ≤ 0.05; **P ≤ 0.01.

The localization of ACE2 was examined in the lungs of mice between PND4 and 2 years of age by immunohistochemistry so as to better understand the temporal spatial pattern of its expression. ACE2 was primarily detected in airway epithelial cells with minimal staining seen in the alveolar space (Fig. 1a). The intensity of ACE2 staining increased steadily in the airway epithelium throughout the life of the mouse. A rare ACE2-positive alveolar cell (arrows) was first observed on PND7 and then steadily increased in number between 6 and 24 months of age. Western blotting for ACE2 confirmed that the abundance of ACE2 protein became progressively enriched in the whole lungs of 12- and 24-month-old mice relative to those of mice harvested at 2 months of age (Fig. 1b). ACE2 mRNA levels were similarly increased in the lungs of 12 and 24-month mice when compared to 2-month mice (Fig. 1c).

**Figure 2:**
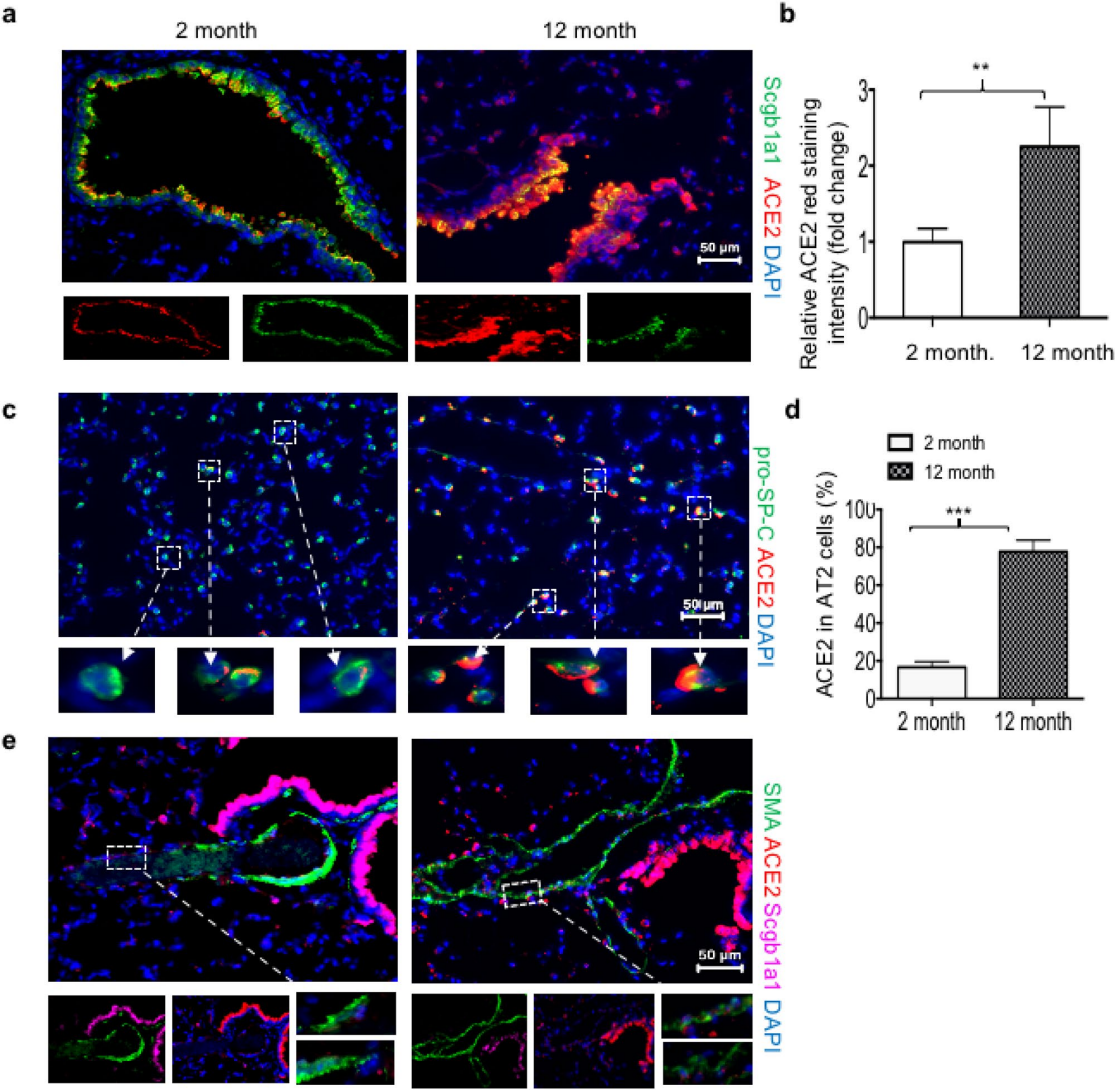
Aging increases ACE2 expression in airway Club and alveolar type 2 cells. (**a**) Lungs from 2-month and 12-month-old mice were immunostained for ACE2 (red), Scgb1a1 (green), and counterstained with DAPI (blue). Boxed sections are individual ACE2 and Scgb1a1 stains. (**b**) Quantification of airway of ACE2 staining was determined from 5 random airway images of 5 mice per group using identical exposure time. Scale bar = 50 μm. (**c**) Lungs were stained for ACE2 (red), proSP-C (green), and counterstained with DAPI (blue). Boxed sections are enlarged below each figure. (**d**) The proportion of proSP-C + cells expressing ACE2 was quantified from 5 random images taken from 5 mice per group and graphed. Statistical significance is comparisons for all pairs using Tukey–Kramer HSD test, with **P ≤ 0.01; ***P ≤ 0.001. Bar = 50 μm.

The cellular source of ACE2 was determined by co-staining ACE2 with antibodies against various cell-specific proteins. Co-staining with antibodies for ACE2 and the Club cell marker secreteglobin1a1 (Scgb1a1) showed extensive co-localization along the airways at both 2 and 12 months of age (Fig. 2a), but the intensity of ACE2 staining was significantly higher at 12 months of age than at 2 months of age (Fig. 2b). Co-staining for ACE2 and the AT2 cell marker proSP-C revealed that the vast majority of ACE2 + cells in the alveoli were AT2 cells (Fig. 2c). Approximately 20% of proSP-C + AT2 cells expressed ACE2 at 2 months while 80% of proSP-C + AT2 cells expressed it at 12 months (Fig. 2d). Less intense ACE2 staining was detected in large arterioles defined by their expression of alpha-smooth muscle actin and lack of expression of Scgb1a1 (Fig. 2e). However, ACE2 staining in these cells did not appreciable change with age. These findings reveal that ACE2 is primarily expressed by the airway Club cells of young adult mice and becomes increasingly expressed by these cells and by alveolar AT2 cells as mice age.

### Neonatal hyperoxia enhances the age-dependent changes in ACE2 expression

**Figure 3:**
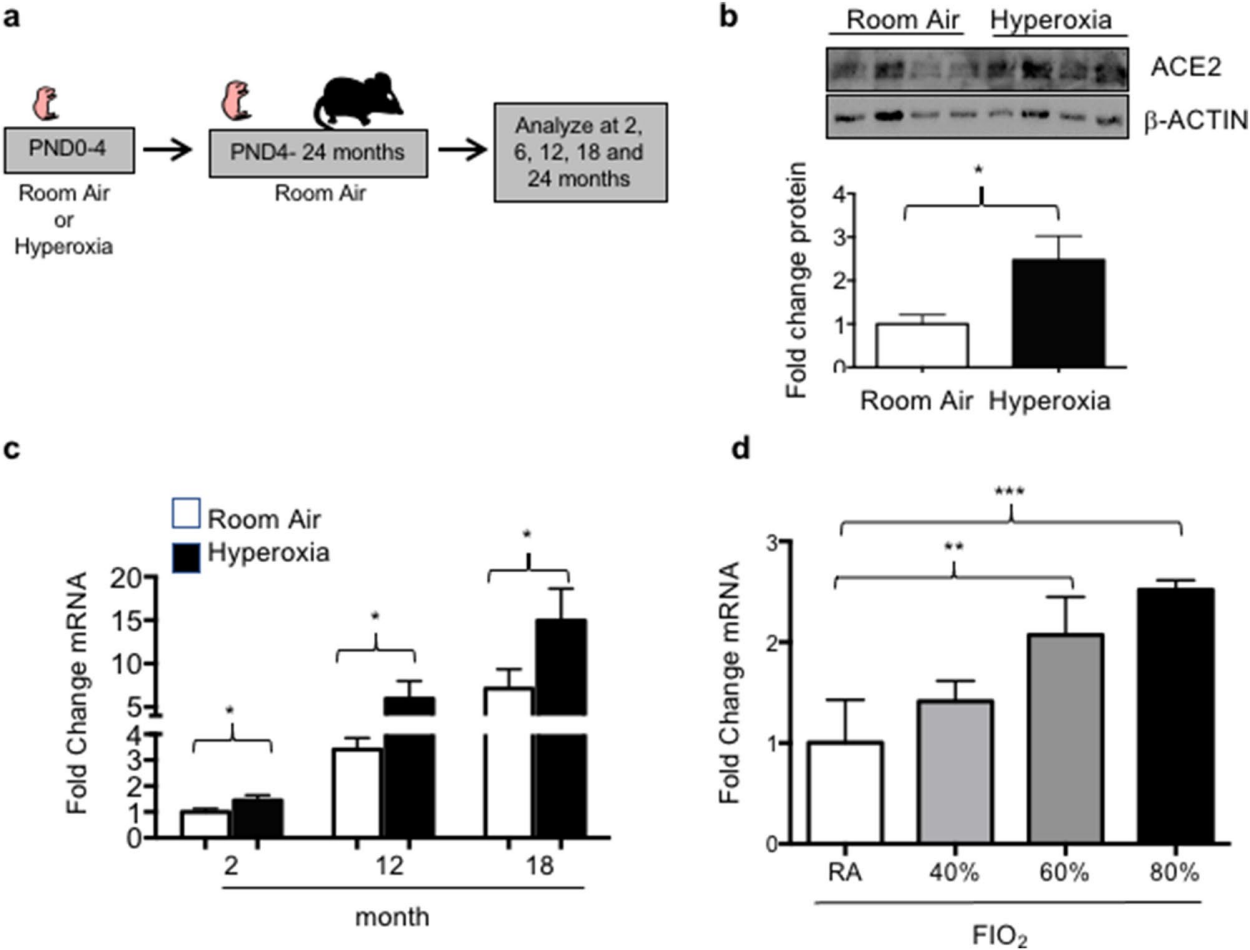
Neonatal hyperoxia stimulates expression of ACE2 in adult mice. (**a**) Cartoon showing the experimental approach of exposing newborn mice to hyperoxia. (**b**) Total lung homogenates were immunoblotted for ACE2 and β-ACTIN as a loading control. Data obtained from 5 mice per group is graphed as mean ± SD fold change over room air values. (**c**) qRT-PCR was used to quantify *Ace2* mRNA in total lung homogenates of 2-, 12-, and 18-month-old mice exposed to room air or hyperoxia between PND0-4. Values were normalized to expression of *18S* RNA and graphed as mean ± SD fold change of ACE2 in 2-month-old room air mice. N = 5 mice per group. (**d**) qRT-PCR was used to quantify *Ace2* mRNA in total lung homogenates of 2-month-old mice exposed to room air, 40%, 60%, or 80% oxygen between PND0-4. Values were normalized to expression of *18S* RNA and graphed as fold change of ACE2 in 2 month room air mice. N = 5 mice per group. Statistical significance for b-d is comparisons for all pairs using Tukey–Kramer HSD test with *P ≤ 0.05; **P ≤ 0.01; ***P ≤ 0.001.

We previously showed that adult mice exposed to 100% oxygen between PND0-4 (Fig. 3a) have 50% fewer AT2 cells than mice exposed to room air^21^and thus expected ACE2 expression to be lower in the lungs of mice exposed to neonatal hyperoxia than in those of controls. It was therefore surprising to find that the levels of ACE2 protein were higher in the lungs of 2-month-old mice that were exposed to neonatal hyperoxia than in age-matched control lungs (Fig. 3b). The levels of *Ace2* mRNA were also increased in the lungs of neonatal hyperoxia-exposed mice at 2 months of age and remained higher than in the lungs of age-matched controls at 6 and 12 months of age (Fig. 3c). To determine the amount of oxygen needed to stimulate the expression of *Ace2*, the lungs of 2-month old mice exposed to 0, 40, 60 or 80% oxygen from PND0-4 were examined by qRT-PCR (Fig. 3d). The levels of *Ace2* expression was significantly higher in adult mice exposed to 60% and 80% oxygen compared to controls exposed to room air. Exposing mice to 40% oxygen for 8 days^22^, which is a larger cumulative dose than 60% oxygen for 4 days also did not increase Ace2 expression (data not shown). We recently deposited an RNA-seq analysis of AT2 cells isolated from PND4 mice exposed to room air or hyperoxia that shows hyperoxia modestly decreases *Ace2* mRNA abundance (Gene Expression Omnibus of the National Center for Biotechnology Information under the accession number GSE140915). Taken together, these findings suggest hyperoxia at birth stimulates expression of ACE2 as mice age.

**Figure 4:**
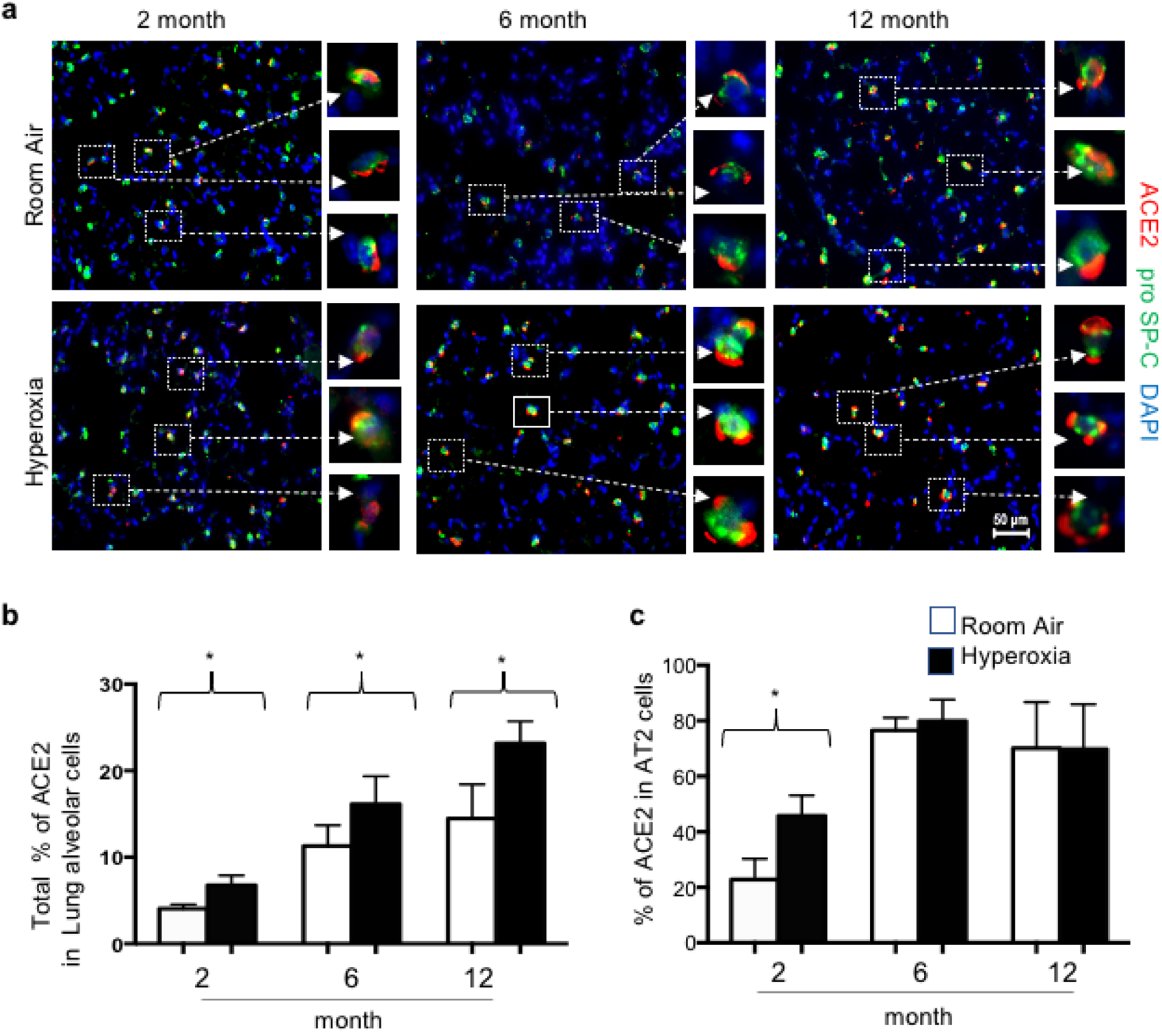
Neonatal hyperoxia stimulates expression of ACE2 in alveolar type 2 cells. (**a**) Lungs of 2-, 6- and 12-month-old mice exposed to room air or hyperoxia between pnd0-4 were stained for ACE2 (red), proSP-C (green), and DAPI. Upper rows reflect room air and lower rows reflect hyperoxia between PND0-4. Boxed regions are enlarged to the right of each image. (**b**) The proportion of ACE2-positive to total DAPI cells was quantified in 5 random fields taken from 5 mice per group and graphed. (**c**) The proportion of proSP-C + cells that express ACE2 were quantified in 5 random fields taken from 5 mice per group and graphed. Values in b and c represent mean ± SD of 4–5 lungs per group. Statistical significance is comparisons for all pairs using Tukey–Kramer HSD test with*P ≤ 0.05.

Immunohistochemistry was used to further understand how hyperoxia affected ACE2 expression in the adult lung. While neonatal hyperoxia modestly increased intensity of ACE2 staining in the airway, it most obviously increased the number of alveolar cells with detectable ACE2 (Fig. 4a). When quantified, neonatal hyperoxia increased the number of alveolar cells expressing ACE2 by approximately 50% at 2, 6 and 12 months of age (Fig. 4b). The increased alveolar expression seen at 2 months of age was primarily attributed to increased expression by proSP-C + AT2 cells; however, this difference resolved at 6 and 12 months of age as more AT2 cells in control lungs began to express ACE2 (Fig. 4c). Hyperoxia did not appreciably change the intensity of ACE2 staining in endothelial cells of larger vessels.

### Scavenging mitochondrial ROS block oxygen-dependent changes in ACE2 and TMPRSS2 expression

**Figure 5:**
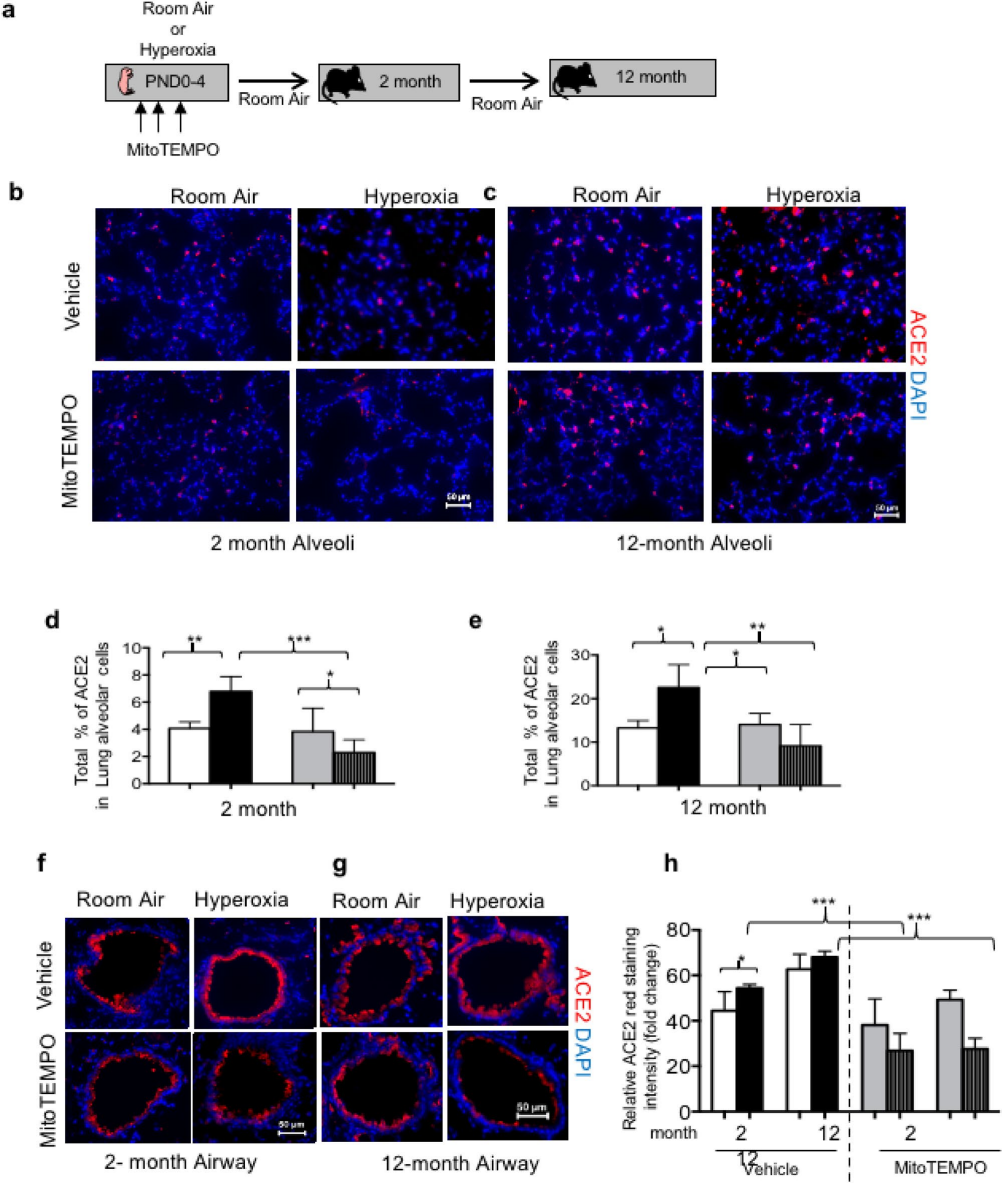
Anti-oxidants prevent hyperoxia from stimulating expression of ACE2. (**a**) Cartoon showing the experimental approach of exposing newborn mice to hyperoxia and treated with mitoTEMPO (PND0-4). (**b**) Alveolar regions of 2-month and 12-month mice were stained for ACE2 (red), and counterstained with DAPI (blue). (**c**) The proportion of ACE2 positive cells to total DAPI positive cells were quantified in 5 random images from 5 mice per group and graphed. (**d**) Airways of 2-month and 12-month mice were stained for ACE2 (red), and counterstained with DAPI (blue). (**e**) Staining intensity of ACE2 was determined from 5 random airway images of 5 mice per group using identical exposure time. Scale bar = 50 μm; Statistical significance is comparisons for all pairs using Tukey–Kramer HSD test with *P ≤ 0.05; **P ≤ 0.01; ***P ≤ 0.001.

Prior studies by us and other investigators found that administering the mitochondrial superoxide scavenger mitoTEMPO to mice during exposure to hyperoxia prevents alveolar simplification and pulmonary vascular disease in adult mice^23–25^. The effect of mitoTEMPO on ACE2 expression was investigated in 2 and 12 month mice who had been exposed to room air or hyperoxia as neonates (Fig. 5a). Neonatal hyperoxia increased alveolar ACE2 staining in lungs of 2 and 12 month mice, which was suppressed by mitoTEMPO (Fig. 5b,c). MitoTEMPO reduced the number of alveolar cells with detectable levels of ACE2 protein at both ages (Fig. 5d,e). It also reduced the intensity of ACE2 staining in airway Club cells seen in 2 month and 12 month mice (Fig. 5f–h).

**Figure 6:**
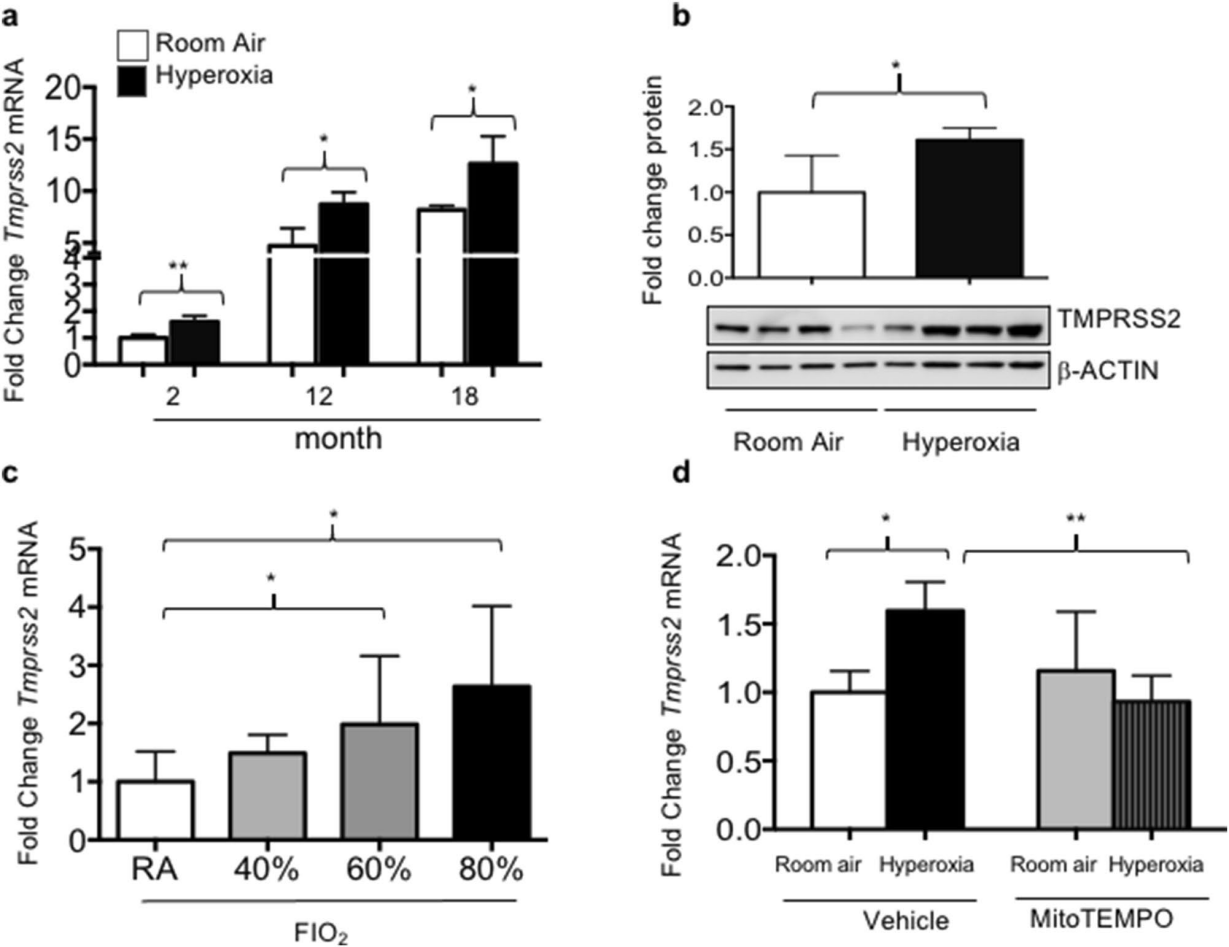
Neonatal hyperoxia stimulates age-dependent expression of *Tmprss2* mRNA. (**a**) qRT-PCR was used to quantify *Tmprss2* mRNA in total lung homogenates of 2-, 12-, and 18-month-old mice exposed to room air or hyperoxia between PND0-4. Values from 5 mice per group were normalized to expression of *18S* RNA and graphed as fold change of ACE2 in 2-month-old room air mice. (**b**) Lungs homogenates prepared from 2-month mice were immunoblotted for TMPRSS2 and β-ACTIN as a loading control. Each lane represents an individual mouse. Band intensity of TMPRSS2 to β-ACTIN was quantified and graphed as fold change relative to 2-month samples. Bars reflect mean ± SD graphed. (**c**) qRT-PCR was used to measure *Tmprss2* mRNA in total lung homogenates of 2 month mice exposed to room air, 40%, 60%, or 80% oxygen between PND0-4. (**d**) qRT-PCR was used to measure *Tmprss2* mRNA in control and 2-month-old mice exposed to room air or hyperoxia and vehicle or mitoTEMPO between PND0-4. N = 4–5 mice per group for b, c. Statistical significance is comparisons for all pairs using Tukey–Kramer HSD test with *P ≤ 0.05; **P ≤ 0.01.

TMPRSS2 is an endoprotease expressed by respiratory epithelial cells and endothelial cells that facilitates viral entry of coronaviruses into epithelial cells^6^. The levels of Tmprss2 mRNA and protein were examined in the lungs of 2-, 12- and 18-month-old mice that were exposed to neonatal hyperoxia and room air from PND0-4 by qRT-PCR and western blotting. *Tmprss2* mRNA was readily detected in the lungs of 2-month mice, and increased ~ fivefold at 12 months and ~ eightfold at 18 months (Fig. 6a). Neonatal hyperoxia further increased *Tmprss2* expression by ~ 50% at each time-point examined. Western blotting similarly showed that the levels of TMPRSS2 protein were higher in the whole lung lysates of 2 month mice exposed to neonatal hyperoxia than in those of control mice (Fig. 6b). As observed for *Ace2* expression, exposure to ≥ 60% oxygen from PND4-0 was required to significantly increase the levels of Tmprss2 mRNA in the lungs of mice at 2 months of age (Fig. 6c). Exposure to 40% oxygen from PND0-8 also failed to change Tmprss2 expression in adult mice (data not shown) while the administration of mitoTEMPO to mice during exposure blunted the effects of neonatal hyperoxia on Tmprss2 mRNA (Fig. 6d). While we were unable to identify the source of TMPRSS2 using commercially available antibodies and immunohistochemistry, our findings reveal age and neonatal hyperoxia increases TMPRSS2 in the lung, as they do for ACE2.

### MitoTEMPO suppresses persistent oxidative stress and DNA damage as mice age

MitoTEMPO scavenges mitochondrial superoxide produced during hyperoxia that could be responsible for directly stimulating expression of ACE2 and TMPRSS2. However, as mentioned earlier, hyperoxia does not directly stimulate *Ace2* in PND4 mice and it does not simulate *Ace2* or *Tmprss2* in adult mice (Supplemental Fig. 1S). Alternatively, mitoTEMPO might blunt oxidative stress and damage resulting in long-term changes in inflammation and vascular injury. However, neonatal hyperoxia did not affect wet/dry lung ratios, a biomarker of vascular injury (Supplemental Fig. 2S) or provoke a persistent inflammatory state in 2 month old mice^16,26^. MitoTEMPO might prevent epigenetic changes in Ace2 and Tmprss2 transcription because hyperoxia for more than 2 weeks increases DNA hypermethylation^27^. But DNA hypermethylation was not seen in our shorter model of 4 days hyperoxia until mice were 1 year old, which is several months after hyperoxia increases expression of *Ace2* and *Tmprss2* (Supplemental Fig. 3S).

**Figure 7:**
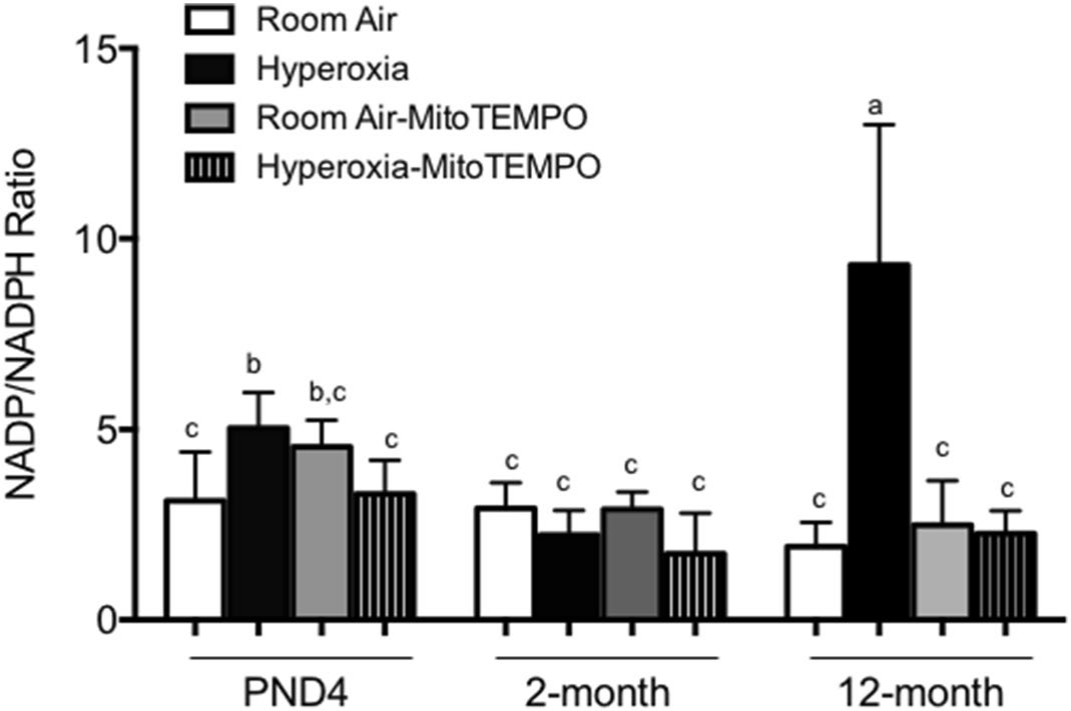
MitoTEMPO blunts NADPH oxidase activity in mice exposed to hyperoxia. The ratio of oxidized NADP and reduced NADPH was measured in homogenized lungs collected from PND4, 2 month, and 12 month mice exposed to room air or hyperoxia and administered mitoTEMPO or vehicle control. Values represent mean + /- SEM ratio of NADP/NADPH per milligram of whole lung tissue collected from 4–5 mice per group. Statistical significance is comparisons for all pairs using Tukey–Kramer HSD test. Bars with the same letter are not significantly different while bars with different letters are significantly different between each other.

Neonatal hyperoxia stimulates expression of p47^phox^ and Nox1, which independently complex with other proteins to respectively form NADPH oxidase 1 and NADPH oxidase 2^24,28^. Furthermore, mitoTEMPO blocked the increased expression of Nox1 seen on PND7^24^. To confirm mitoTEMPO inhibits the oxygen-dependent activation of NADPH oxidase activity, we evaluated NADPH oxidase activity in the lung by measuring the ratio of oxidized NADP to reduced NADPH. Hyperoxia increased the ratio of oxidize to reduced NADPH in PND4 mice, which was suppressed by mitoTEMPO (Fig. 7). At 2-months, the ratio of NADP to NADPH was comparable in mice exposed to room air or hyperoxia as neonate, and not affected by mitoTEMPO. To our surprise, the ratio of oxidized NADP to reduced NADPH was markedly elevated in lungs of 12 month mice exposed to hyperoxia as neonates, which was dampened by administering mitoTEMPO during exposure. While additional studies are required to determine when and where these changes occur, it is clear that mitoTEMPO blocks oxygen-dependent changes in NADPH oxidation as mice age.

**Figure 8:**
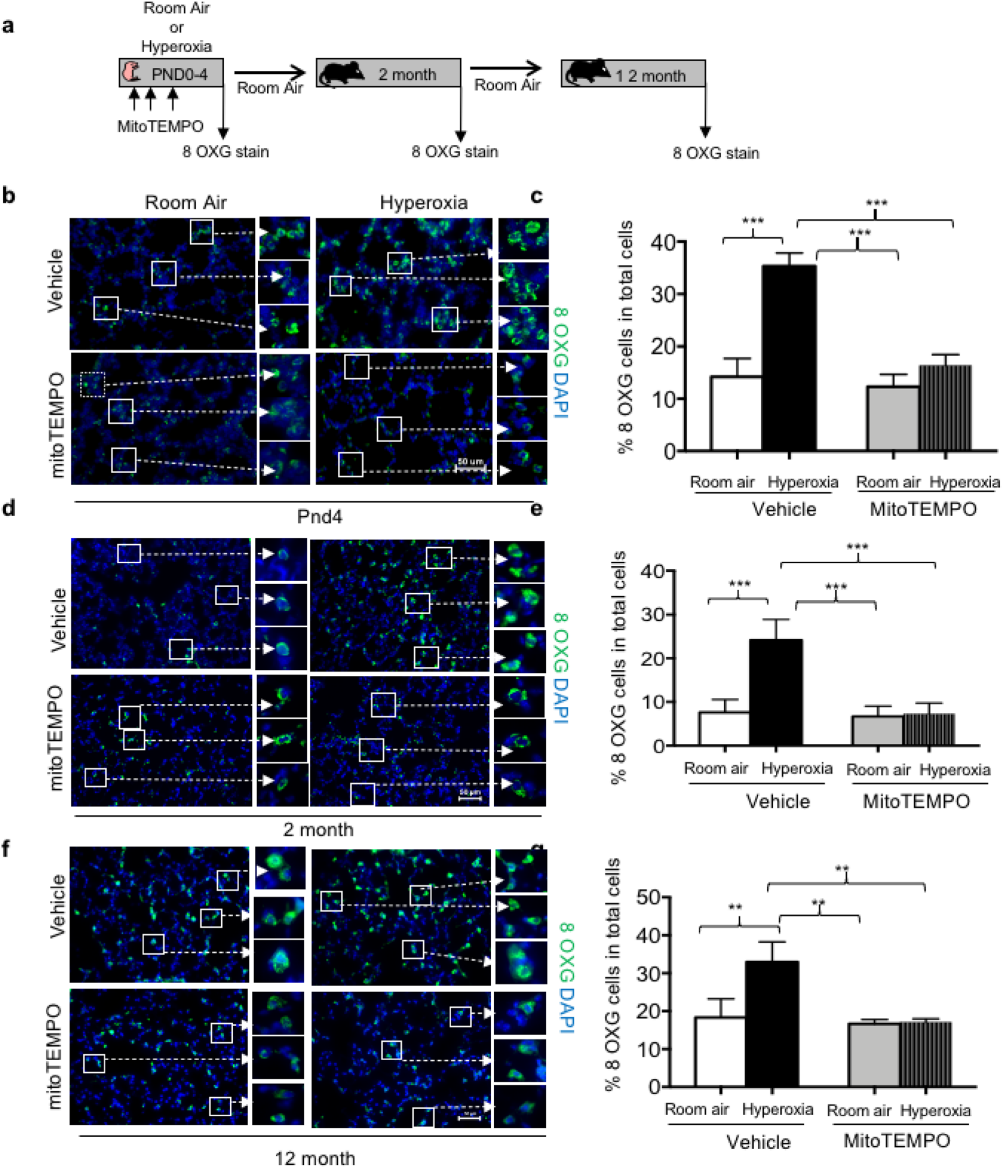
MitoTEMPO blunts oxidative DNA damage in mice exposed to hyperoxia. (**a**) Cartoon model showing when 8-oxoguanine staining was investigated in PND4 mice exposed to room air or hyperoxia and administered mitoTEMPO or vehicle. Lungs of PND4 (**b**), 2 month (**d**), and 12 month (**f**) mice exposed to room air or hyperoxia as neonates were stained with for 8-oxoguanine (green) and counterstained with DAPI (blue). Boxed regions are enlarged to the right of each image. The proportion of 8-oxoguanine + to total DAPI + cells in PND4 (**c**), 2 month (**e**), and 12 month mice (**g**) mice were quantified in 5 random images taken from 5 mice per group and graphed. Values represent mean ± /SEM. Statistical significance is comparisons for all pairs using Tukey–Kramer HSD test with **P ≤ 0.01, ***P ≤ 0.001.

We and others have shown how hyperoxia increases 8-oxoguanine staining in airway and alveolar epithelial cells^29,30^. This staining reflects oxidation of mitochondrial DNA because it was cytoplasmic and colocalized with mitochondrial-specific cytochrome c oxidase subunit 1. MitoTEMPO might prevent the oxidation of mitochondrial DNA during hyperoxia because it is a mitochondrial-targeted superoxide mimetic. Indeed, neonatal hyperoxia increased cytoplasmic 8-oxoguanine staining in both airway and alveolar cells of PND4 mice that was reduced by mitoTEMPO (Fig. 8a–c). Interestingly, increased 8-oxoguanine staining seen in PND4 mice exposed to hyperoxia was still evident at 2 (Fig. 8d,e) and 12 months (Fig. 8f,g) of age. Moreover, it was diminished in mice who were administered mitoTEMPO during hyperoxia. These findings extend prior studies showing that mitoTEMPO blunts oxygen induced ROS by now showing that it also suppresses oxygen-induced oxidative DNA damage that persists long after mice had returned to room air.

## Discussion

The COVID-19 outbreak was first detected in the Chinese city of Wuhan in 2019 and has since expanded rapidly to become one of the worst pandemics to ever challenge the modern world. While people of all ages are susceptible to infection, the severity of disease is worse in people who are elderly or who have pre-existing health conditions including COPD, diabetes, hypertension, and cancer^29^. Those with multiple co-morbidities have a higher rate of mortality. People born preterm may also be at great risk for COVID-19 because they often suffer from multiple co-morbidities due, in part, to their lungs being exposed to oxygen too soon or to super-physiological concentrations used to maintain appropriate blood oxygen saturations. It is unclear whether co-morbidities increase disease by changing spatial and temporal expression of SARS-CoV-2 receptors or the immune response that leads to a lethal cytokine storm^1^. In this study, we present evidence that expression of the SARS-CoV-2 co-receptors ACE2 and TMPRSS2 increase in the lungs of mice as they age and this can be stimulated or accelerated by early exposure to hyperoxia. Increased expression of ACE2 found in distal AT2 cells was of particular interest because infection of these cells with other viruses has been associated with higher mortality in humans^12–14^. When infected by influenza A virus, AT2 cells may contribute to lung disease by producing inflammatory molecules that contribute to a lethal cytokine storm^9^. They may also die and therefore reduce the number of surviving AT2 cells required to serve as stem cells for alveolar regeneration^20,30,31^. Our findings support the idea that age and co-morbidities like preterm birth may increase the severity of COVID-19 by changing temporal and spatial patterns of SARS-CoV-2 receptors.

We found that ACE2 was primarily expressed by airway Club cells during early postnatal life. The intensity of ACE2 staining increased in the airways of mice with age and became detectable in the alveoli of young adult mice. Co-localization with proSP-C revealed that most, but not all alveolar cells expressing ACE2 were AT2 cells. Our findings are consistent with an earlier study showing that ACE2 is expressed in the adult mouse lung by Clara cells (now called Club cells), AT2 cells, and to some extent by endothelial cells around small and medium sized vessels^32^. While that study showed how ACE2 levels rise during fetal development, our findings extend it by showing that ACE2 expression continues to increase as mice age. We also found that *Tmprss2* mRNA expression increases as mice age and this expression was similarly enhanced by neonatal hyperoxia. While AT2 cells have previously been shown to express TMPRSS2^8^, we were not able to reliably detect it in the mouse lung using commercially available antibodies. However, we did find that the abundance of *Tmprss2* mRNA and protein abundance increased with age and neonatal hyperoxia, and was reduced by mitoTEMPO similar to that of *Ace2*. The higher expression of these genes as mice age is in agreement with recent review that discussed two unpublished studies deposited in *bioRxiv* showing how expression of *Ace2* and *Tmprss2* mRNA increases with age in human respiratory epithelium^1^. Those findings in humans and ours in mice suggest the age-dependent increase in SARS2-CoV-2 receptors may be responsible for increasing the severity of COVID-19 lung disease in elderly people.

It is important to discuss the normal functions of ACE2 and TMPRSS2 because that may help explain why their expression steadily increases with age^33^. ACE2 is perhaps best known for its role in controlling blood pressure in the renin-angiotensin system^34^. ACE1 converts the 10-amino acid angiotensin I to an 8-amino acid vasoconstrictive peptide called angiotensin II. ACE2 accumulates in people with pulmonary hypertension and hydrolyzes Angiotensin II to Ang(1–7), which has vasodilation properties. Over-expressing ACE2 also protects against right ventricular hypertrophy^35^. Hence, higher levels of ACE2 seen as the lung ages may reflect an adaptive response designed to protect against the development of cardiovascular disease. Interestingly, ACE2 levels decline in bleomycin-induced lung fibrosis and humans with interstitial pulmonary fibrosis while angiotensin II levels rise^36,37^. Angiotensin II can promote fibrosis by stimulating AT2 cell apoptosis downstream of TGF-β signaling^38^. ACE2 serves as an anti-fibrotic molecule by stimulating the hydrolysis of angiotensin II to Ang(1–7), which in turn signals through the Mas oncogene to block AT2 cell apoptosis by suppressing JNK activation^39^. The slow and steady increase in ACE2 expression as the lung ages may also serve to preserve AT2 cells and thus reduce or prevent the development of idiopathic pulmonary fibrosis. In contrast to ACE2, TMPRSS2 is a serine protease that is localized to the apical surface of secretory cells such as Club and AT2 cells of the lung^40^. It is difficult to speculate on why TMPRSS2 expression increases with age because its role in lung development, homeostasis, and repair is poorly understood.

Our study also found that neonatal hyperoxia increased or accelerated expression of *Ace2* mRNA, ACE2 protein, and *Tmprss2* mRNA as mice age. Significant changes were seen with 60% or more FiO_2_ at 8 weeks (2 months) of age and persisted as mice age. How hyperoxia regulates expression of these proteins is conflicting and remains to be better understood. One study using human fetal IMR-90 fibroblasts found that hyperoxia does not change expression of ACE2^41^. However, ACE2 was depleted shortly after cells returned to room air presumably because it was being proteolyzed and shed into the media. In contrast, another study found higher levels of ACE2 in newborn rats exposed to 95% oxygen for the first week of life and then recovered in 60% oxygen for the next 2 weeks^42^. In our hands, changes in *Ace2* or *Tmprss2* mRNA were not seen at PND4 but rather were first detected when mice were ~ 8 weeks old. In fact, we recently deposited an RNA-seq analysis of AT2 cells isolated from PND4 mice exposed to room air versus hyperoxia that shows hyperoxia modestly inhibits *Ace2* and increases *Tmprss2* mRNA abundance (Gene Expression Omnibus of the National Center for Biotechnology Information under the accession number GSE140915). Exposing adult mice to hyperoxia also did not stimulate their expression in the lung. This is an important observation because it suggests oxygen therapies used to treat people with COVID19 might not change receptor expression and thus would not accelerate infection. Hyperbaric oxygen therapies use high pressure to increase blood oxygen saturation and enhance oxygen delivery outside the lung. It is used to treat cancer and wounds that don’t heal. Our finding that hyperoxia does not affect expression of ACE2 in pulmonary arterioles suggest hyperbaric oxygen therapies might not affect ACE2 expression either. Taken together, our findings strongly indicate neonatal hyperoxia does not directly affect expression of ACE2 and TMPRSS2 until after the mice are returned to room air. Because ACE2 and TMPRSS2 were only affected by doses of oxygen that cause long-term changes in lung function (i.e., 60% for 4 days but not 40% for 4 or 8 days), we speculate that they occur as an adaptive response to oxygen as mice age. The elevated expression of ACE2 and perhaps TMPRSS2 may serve to prevent the loss of AT2 cells damaged by early oxygen and promote vasodilation as the pulmonary capillary bed undergoes rarefaction^21,43^. But higher levels of these proteins may become a maladaptive response if they render the lung more susceptible to coronavirus infections.

The early expression of ACE2 and TMPRSS2 seen in adult mice exposed to neonatal hyperoxia was inhibited by administering mitoTEMPO during exposure. Since mitoTEMPO blocks oxygen-dependent expression of Nox1^24^, we measured NADPH oxidase activity as a surrogate downstream mediator of mitochondrial ROS responses. As expected, neonatal hyperoxia increased NADPH oxidase activity, which was suppressed by mitoTEMPO. Increased NADPH oxidase activity seen during hyperoxia declined when mice were returned to room air such that there was no difference detected in 2 month mice. But surprisingly, NADPH oxidase activity was markedly higher in 12 month mice exposed to hyperoxia as neonates when compared to controls exposed to room air. This increased and persistent oxidation may damage the lung worsening respiratory health as mice age. Consistent with this idea, we investigated and found that neonatal hyperoxia increased cytoplasmic 8-oxogunaine staining in airway and alveoli, which has been shown to reflect oxidation of mitochondrial DNA^29,30^. Unexpectedly, 8-oxoguanine also persisted into adulthood suggesting that mitochondrial DNA repair was not efficient or mitochondrial DNA is continuously damaged perhaps as NADPH oxidase activity increases with age. While additional studies are needed to identify the cellular source of NAPDH oxidase activity and its role in oxidizing DNA, these findings provide exciting new evidence that early life mitochondrial oxidative stress primes the lung for enhanced oxidation and DNA damage later in life. Whether this directly or indirectly contribute to the expression of SARS-CoV-2 receptor expression remains to be determined. Anti-oxidant therapies may therefore prove useful for suppressing expression of SARS-CoV-2 receptors and reducing the severity of COVID-19 related lung disease in people with pre-existing co-morbidities caused by chronic oxidative stress. There is precedence for this idea because transgenic mice that over-express extracellular superoxide dismutase in AT2 cells develop less lung disease when infected with influenza A virus than controls^44^.

Increased expression of ACE2 and TMPRSS2 may not be the only way these proteins enhance the severity of COVID-19-related lung disease. For example, TMPRSS2 facilitates viral activation and entry by cleaving hemagglutinin on influenza A virus and the spike protein on the SARS-CoV-2 virus^8^. The spike protein accesses the cell when it binds the glucose regulated protein 78 (Grp78, BiP) found on the cell surface^45^. Grp78 is a master regulator of the unfolded protein response (UPR)^46^. It is normally localized to the endoplasmic reticulum (ER) where it inhibits the UPR by binding Activating Transcription Factor 6 (ATF6), Protein kinase RNA-like Endoplasmic Reticulum Kinase (PERK), and Inositol-requiring Enzyme 1 (IRE1). Grp78 is released from these proteins when oxidation and other stressful conditions cause an accumulation of unfolded proteins. It can then escape the ER and traffic to the cell surface where it becomes available to bind the coronavirus S protein and facilitate viral entry. This information should raise great concern for people with familial forms of IPF caused by mutations in *SFTPC* and other genes that activate the UPR in AT2 cells^47^. Genetic studies in mice suggest mutant forms of SP-C that activate the UPR are not sufficient by themselves to cause fibrotic lung disease. However, they can predispose the lung to fibrotic disease following viral infections^48^. Familial forms of IPF that activate the UPR in AT2 cells may therefore accelerate the age-dependent susceptibility of AT2 cells to SARS-CoV-2 infections.

There are several limitations to our study. First, we were not able to confirm mitoTEMPO blunts mitochondrial ROS in live AT2 cells isolated from mice because COVID19 prevented us from accessing equipment needed for this measurement. Instead, we provided new and compelling supportive evidence that mitoTEMPO blunted oxygen-induced DNA damage and downstream NADPH oxidase activity. Second, although neonatal hyperoxia does not cause vascular leak (Supplemental Fig. 2S) or inflammation^16,26^ in 2 month old mice, it remains to be determined whether this changes as mice become elderly, and whether that contributes to SARS-CoV-2 receptor expression. Third, we cannot rule out that persistent DNA damage or oxidative stress causes epigenetic changes that drive Ace2 and Tmprss2 expression as mice age. Finally, we could not reliably identify cells expressing TMPRSS2 using commercially available antibodies and immunohistochemistry. *Tmprss2* mRNA has been detected in AT2 cells isolated from mice (Gene Expression Omnibus of the National Center for Biotechnology Information under the accession number GSE140915). However, recent studies indicate expression of *Tmprss2* and *Ace2* mRNA is higher in lungs of adult humans than children^1,49^. According to publicly available transcriptomic datasets of nasal and lung airway tissue, adult humans and smokers have higher levels of *Tmprss2* in their upper and lower airways than children or non-smokers^50^. Tobacco smoke and hyperoxia may stimulate *Tmprss2* though similar mechanisms because they both damage DNA and cause persistent oxidative stress.

In summary, we found that neonatal hyperoxia increases or accelerates the age-dependent expression of ACE2 and TMPRSS2 in the mouse lung. Understanding how expression of these proteins changes with age and in response to early life insults such as neonatal hyperoxia may provide new opportunities for reducing the severity of COVID-19 and other types of lung disease.

## Materials and methods

### Mice

C57BL/6 J mice were purchased from the Jackson Laboratories and maintained as an inbred colony. Mice were exposed to room air (21% oxygen) as control or hyperoxia (100% oxygen unless otherwise stated) between birth and postnatal day (PND) 4 and then returned to room air^17^. Dams were cycled every 24 h to ensure that hyperoxia did not compromise their health. Some mice exposed to room air or hyperoxia were injected intraperitoneally with 0.7 μg/g mitoTEMPO (Enzo Life Sciences, Farmingdale, NY) or vehicle (phosphate-buffered saline) on PND0, PND1, and PND2. All mice used in this study were of mixed sex and housed in a pathogen-free environment according to a protocol (UCAR2007-121E) approved by the University Committee on Animal Resources at the University of Rochester. All experiments performed with mice were in accordance with the relevant guidelines and regulations of this committee.

### Immunohistochemistry

Mice were euthanized with 100 mg/kg sodium pentobarbital injected I.P. The chest was exposed and the lungs were inflation fixed overnight via the trachea in 10% neutral buffered formalin, embedded in paraffin and 4 mm sections prepared^21,51^. Sections were stained with antibodies against ACE2 (25ug/ml Invitrogen, PA5-47,488, Waltham, MA), Scgb1a1 (1:500, Sigma, 07–063, St. Louis, MO) and proSP-C (1:1000, Seven Hills Bioreagents, Cincinnati, OH). Immune complexes were detected with fluorescently labeled secondary antibody (1:200, Jackson Immune Research, West Grove, PA). For 8-oxoguanine staining, lungs were incubated overnight with FITC-conjugated 8-oxoguanine binding peptide using OxyDNA assay kit (Calbiochem, 500,095, San Diego) as previously described^52^. Sections were covered in mounting medium containing 4′, 6-diamidino-2-phenylindole (DAPI) (SouthernBitotech 0100–20, Birmingham AL ) before viewing with Nikon E800 Fluorescence microscope (Microvideo Instruments, Avon, MA) and a SPOT-RT digital camera (Diagnostic Instruments, Sterling Heights, MI). Approximately 4–5 images at 40X were captured from each lung and positive stained cells or nuclei were counted. For each stain, the proportion of positively stained DAPI-positive cells was quantified and averaged over all of the images taken from an individual lung. This value was then used to calculate the mean average value with SEM for all of the lungs of a given experimental group and graphed.

### Airway Fluorescent intensity measurement

Fluorescently stained slides were imaged by microscopy using the same image settings. Five random images containing distal airway segments were captured and staining intensity of the airway was analyzed with Image J (National Institutes of Health, Bethesda, MD). Data are presented as mean fluorescent intensity per square micron.

### Quantitative RT-PCR

Total RNA was isolated from the lung using Trizol reagent (ThermoFisher Scientific) and reverse transcribed to cDNA using the iScript cDNA synthesis kit (Bio-Rad Laboratories, Hercules, CA). The cDNA was then amplified with SYBR Green I dye on CFX96™ or CFX384™ Real-Time PCR detection system (Bio-Rad Laboratories, Hercules, CA). PCR products were amplified with sequence-specific primers for mouse *Ace2* (sense 5′-GGATACCTACCCTTCCTACATCAGC-3′ and antisense CTACCCCACATATCACCAAGCA-3′), *Tmprss2* (sense 5′- TACTTGGAGCGGACGAGGAA-3′, and antisense 5′- AGGAGGTCAGTATGGGGCTT-3′) or 18S rRNA (sense 5-CGGCTACCACATCCAAGGAA-3′, and antisense 5′- GCTGGAATTACCGCGGCT- 3′) used to normalize equal loading of the template cDNAs. Amplifications were conducted with iTaq Universal SYBR Green Master Mix (Bio-Rad Laboratories, Hercules, CA). Fold changes in gene expression were calculated by the ∆∆Ct method using the Ct values for the housekeeping 18S rRNA as a control for loading.

### Western blot analysis

The left lung lobe was homogenized in lysis buffer and insoluble material removed by centrifugation^21^. Equal amounts of protein were separated on sodium dodecyl sulfate polyacrylamide gels and transferred to nylon membranes. The membranes were immunoblotted with primary antibodies to ACE2 (2.5ug/ml, Invitrogen, PA5-47488, Waltham, MA), TMPRSS2 (1:1000, Abcam, ab92323, Cambridge, MA) or β-ACTIN (Sigma, A2066). The blots were then incubated in appropriate secondary antibody (Southern Biotech, Birmingham, AL). Immune complexes were detected by chemiluminescence and visualized with a ChemiDoc Imaging System (Bio-Rad Laboratories, Hercules, CA).

### NADP/NADPH oxidase ratio

Flash frozen lungs were homogenized in NADP/NADPH extraction buffer using a commercially available NADH oxidase kit (Biovision, catalog K347-100), Milpitas, CA). Insoluble material was removed by centrifugation and the extracted supernatant was aliquoted into multiple samples. Total NADP and NADPH was measured in one aliquot by incubating in NADP cycling buffer with cycling enzyme mix, and measuring at OD_450nm_. The total amount of NADPH was measured in another aliquot after NADP was removed by incubating the sample at 60C for 30 min. The ratio of NADP/NADPH was calculated as (NADPt – NADPH/NADPH where NADPt = NADP/NADPH.

### Statistical analysis

Data were evaluated using JMP14 software (SAS Institute, Cary, NC) and graphed as means ± SEM. An unpaired t-test and 2-way ANOVA were used to determine overall significance, followed by Tukey–Kramer HSD tests. The number of samples per analysis are defined in the figure legend.

## Supplementary Information

Supplementary Information

## Data Availability

The datasets generated during and/or analyzed during the current study are available from the corresponding author on reasonable request. Changes in gene expression during hyperoxia were compared to an RNA-seq analysis of AT2 cells isolated from PND4 mice exposed to room air or hyperoxia were previously deposited under Gene Expression Omnibus of the National Center for Biotechnology Information with the accession number GSE140915.
