# Supplementary material for: Neonatal hyperoxia enhances age-dependent expression of SARS-CoV-2 receptors in mice
Source: Sci Rep. 2020 Dec 28;10:22401. doi: 10.1038/s41598-020-79595-2 (PMC7769981; doi:10.1038/s41598-020-79595-2)
Supplement: Supplementary file 1 — Supplementary Information [file 41598_2020_79595_MOESM1_ESM.docx]

**SUPPLEMENTARY DATA**

**Neonatal hyperoxia enhances age-dependent expression**

**of SARS-CoV-2 receptors in mice**

Min Yee^1^

E. David Cohen^1^

Jeannie Haak^1^

Andrew M. Dylag^1^

Michael A. O’Reilly^1,2^

The Department of Pediatrics

School of Medicine and Dentistry,

The University of Rochester,

Rochester, NY 14642

**Materials and Methods**

*Measurement of lung wet/dry ratio.* Lungs of 8-10 week old adult mice exposed to room air or hyperoxia between PND0-4 were harvested. The lung was weighed and then dried in an oven at 65 °C for 72 h to obtain a dry weight. The dried lung was then weighed. The wet/dry lung ratios were then calculated from mixed groups of male and female mice.

*Exposure of adult mice to hyperoxia*. Adult 2 months old mice were exposed to room air or 100% oxygen for 60 hours. Lungs were then harvested for isolation of mRNA used to evaluate expression of *Ace2* and T*mprss2* by qRT-PCR. Both males and females were used for this experiment.

*Quantitation of DNA methylation*. Total genomic DNA was isolated from lungs of 2 and 12 month mice who were exposed to room air or hyperoxia between PND0-4 using the E.Z.N.A. Tissue DNA kit (Omega BIO-TEK, D3396-01, Norcross, GA). DNA with absorbance ratio of 260/280 > 1.6 was used to assess DNA methylation status using the Global DNA Methylation Assay kit (Abcam, ab233486, Cambridge, MA). Total DNA (100ng) from each sample was incubated with 100μl of binding solution in an ELISA plate, covered with plate seal, and incubated at 37°C for 60 minutes according to manufacturer directions. Non-specific complexes were removed by washing in buffer and specific interactions with antibody to 5-methylcytosine was detected with secondary antibody at 450 nm. Staining intensities were quantified against a standard curve provided by the manufacturer and graphed as the percent of 5-methylcytosine over total input DNA.

**Supplement Figure 1S**. Exposing adult mice to hyperoxia does not change abundance of *Ace2* or *Tmprss2* mRNA in the lung. (**a**) Adult mice (2-months) were exposed to room air or 100% oxygen for 60 hours. Total RNA was isolated from their lungs and used to quantify mRNA abundance of *Ace2* (**b**) and *Tmprss2* (**c**) by qRT-PCR. Data is graphed as mean ± S.E.M. fold change relative to room air values. N = 5 per group, (**a**) p=0.4722; (**b**) p=0.153 relative to room air.


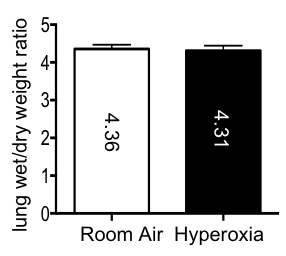


**Supplemental Figure 2S**. Neonatal hyperoxia does not promote edema in the adult mouse lung. Lungs of 2-month old adult mice exposed to room air or hyperoxia between postnatal days 0-4 were weighed. The lungs were then dried at 65$^{\circ}$C for 3 days and then re-weighed. The mean wet-to-dry lung weight ratios were calculated and graphed as mean ± S.E.M. (n= 5 mice per group). The mean values are stated with each bar. P = 0.5535.


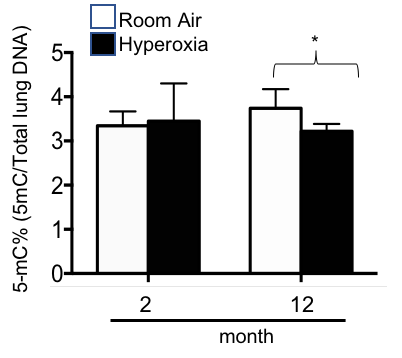


**Supplemental Figure 3S**. Neonatal hyperoxia promotes DNA hypomethylation as mice age. Genomic DNA was isolated from lungs of 2 month and 12 month old mice who had been exposed to room air or hyperoxia between postnatal days 0-4. The amount of 5-methylcytosine was detected in 100 ng of DNA by ELISA and quantified against a standard. The mean levels of 5-methylcytosine ± S.E.M. were quantified and graphed (n = 5 per group). Neonatal hyperoxia reduced the amount of DNA methylation detected in 12 month but not 2 month old mice. *P < 0.05 relative to room air at 12 months of age.
